# Continuous Separation of Circulating Tumor Cells from Whole Blood Using a Slanted Weir Microfluidic Device

**DOI:** 10.3390/cancers11020200

**Published:** 2019-02-10

**Authors:** Yousang Yoon, Jusin Lee, Moonsoo Ra, Hyeokshin Gwon, Seungwon Lee, Min Young Kim, Ki-Chun Yoo, Onejae Sul, Chul Geun Kim, Whoi-Yul Kim, Jea-Gun Park, Su-Jae Lee, Young Yiul Lee, Ho Soon Choi, Seung-Beck Lee

**Affiliations:** 1 Department of Electronic Engineering, Hanyang University College of Engineering, 222 Wangsimni-ro, Seongdong-gu, Seoul 04763, Korea; ysyoon88@hanyang.ac.kr (Y.Y.); jusin19@hanyang.ac.kr (J.L.); ravicmoon@gmail.com (M.R.); ghyeokshin@gmail.com (H.G.); sw915281@gmail.com (S.L.); wykim@hanyang.ac.kr (W.-Y.K.); parkjgl@hanyang.ac.kr (J.-G.P.); 2 Department of Life Science and Research Institute for Natural Sciences, Hanyang University College of Natural Sciences, 222 Wangsimni-ro, Seongdong-gu, Seoul 04763, Korea; 5718my@naver.com (M.Y.K.); vanity0706@gmail.com (K.-C.Y.); cgkim@hanyang.ac.kr (C.G.K.); sj0420@hanyang.ac.kr (S.-J.L.); 3 Institute of Nano Science and Technology, Hanyang University, 222 Wangsimni-ro, Seongdong-gu, Seoul 04763, Korea; ojsul@hanyang.ac.kr; 4 Department of Internal Medicine, Hanyang University College of Medicine, Hanyang University Medical Center, 222 Wangsimni-ro, Seongdong-gu, Seoul 04763, Korea; leeyy@hanyang.ac.kr (Y.Y.L.); hschoi96@hanyang.ac.kr (H.S.C.)

**Keywords:** circulating tumor cell, cell separation, microfluidics

## Abstract

The separation of circulating tumor cells (CTCs) from the peripheral blood is an important issue that has been highlighted because of their high clinical potential. However, techniques that depend solely on tumor-specific surface molecules or just the larger size of CTCs are limited by tumor heterogeneity. Here, we present a slanted weir microfluidic device that utilizes the size and deformability of CTCs to separate them from the unprocessed whole blood. By testing its ability using a highly invasive breast cancer cell line, our device achieved a 97% separation efficiency, while showing an 8-log depletion of erythrocytes and 5.6-log depletion of leukocytes. We also developed an image analysis tool that was able to characterize the various morphologies and differing deformability of the separating cells. From the results, we believe our system possesses a high potential for liquid biopsy, aiding future cancer research.

## 1. Introduction

Circulating tumor cells (CTCs) are cells that are shed from the primary tumor, in the early stage of tumor formation and growth, that circulate through the bloodstream forming secondary tumors on distant organs. They are very rare in the bloodstream; a single tumor cell could be surrounded by a billion background blood cells. However, once properly separated from a patient, CTCs can provide a variety of crucial information regarding cancer and its metastasis [1]. The number of CTCs can be utilized in early detection, and in the prognosis of cancer and real-time monitoring of the effectiveness of therapy [2,3]. Furthermore, when viable CTCs are retrieved with a high purity, their molecular characterization can assist therapeutic selection and scheme to realize personalized treatments for the patient [1,2,3].

The most common approach for separating CTCs, which has shown prognostic value in some cancer types, is by using an antibody that targets the tumor cell specific surface antigens, such as epithelial cell adhesion molecule (EpCAM) [4,5,6,7,8]. However, this method shows limited efficiency, because tumor cells express various levels of epithelial markers because of the epithelial-to-mesenchymal transition (EMT) [9,10,11]. The results imply that the antibody-based positive enrichment of CTCs may lose distinct sub-populations that possess crucial information regarding metastasis, and lead to a biased conclusion [2]. In order to overcome the limitation, other antibodies that are specific to a certain cancer types [12,13,14,15], or the use of a cocktail of tumor specific antibodies, have been reported [16]. However, these methods require information regarding the specific target cancer type or mutation beforehand, or require costly imaging equipment and reagents to visualize the antibody cocktail.

Alternative methods have been developed using distinct physical features of CTCs. One strategy is to utilize their distinctive size; it has been proven that CTCs generally show a greater size than hemocytes [17]. A typical approach of size-based separation is by utilizing filters [18,19,20,21,22,23]. The filtration devices are generally simple and robust, showing a high separation efficiency with a high accessibility to automation [22,23]. However, the hydraulic pressure geared for efficient CTC separation become easily unbalanced as a result of the accumulation of filtered cells, causing the separated CTCs to squeeze through the filters; the capturing of background cells; and, even worse, device failure, owing to filter clogging. Furthermore, persistent contact of the separated cells to the filter gives a high chance of non-specific adhesion. This immobilization hinders the downstream analysis, where the retrieval of filtered cells is required. To overcome these issues, modified filters that allow consistent fluid flow have been reported [24,25,26]. Apart from using filters, CTC separation techniques using hydrodynamics and interactions with external sources have also been introduced. The techniques provide the differential behavior of CTCs induced by spiral channels [27,28,29,30], series of pinched channels or angled trenches [31,32,33,34], an array of pillars [35,36,37], micro-vortexes [38], and acoustic waves [39,40,41].

Even with the variety of CTC separation methods, they depend solely on the size difference suffering from the size overlapping of CTCs with leukocytes [42,43,44]. This impedes the separation performance, forcing a trade-off between efficiency and selectivity. One possible solution to this limitation is to include the CTC’s deformability feature in addition to the size-based separation. Tumor cells are known as being stiffer than leukocytes [45,46], which might be due to the greater nucleus-to-cytoplasm ratio [44,47,48]. Methods to incorporate a deformability-based separation mechanism have been reported using viscoelastic behavior in inertial microfluidics [49], deterministic lateral displacement [50], acoustic sorting [40], and alternating fluid flow [51,52]. However, the utilization of the cell deformability should be performed cautiously and precisely, as tumor cells with a high metastatic potential, those that have undergone EMT, were reported as being more flexible than the less invasive epithelial tumor cells [46,53,54]. 

Here, we present a microfluidic device that is capable of physically separating CTCs from the unprocessed whole blood, continuously, without clogging. The separation was performed by a slanted weir with a gap at the top, which continuously transferred the larger target cells in the blood sample stream to the co-flowing buffer stream, allowing the smaller hemocytes to flow through the gap. Devices with a similar concept, in which the diagonal barrier performs continuous tumor cell separation, have been reported [55,56]. However, without considering the cell deformability, they showed a relatively low purity of the separated tumor cells, or required the binding of microparticles tagged with a tumor specific marker to overcome the size overlapping of the tumor cells with leukocytes. In our microfluidic device, whole blood is accessible and, most importantly, the pressure distribution near the slanted weir is engineered, enabling the precise utilization of the cell deformability, and thus, has a higher rate of leukocyte removal and CTC separation. We analyzed the pressure distribution according to the device geometry by using computational fluid dynamics, and made correlations based on the experimental results. To better optimize for tumor cells with a high metastatic potential, thus being relatively flexible, the experiments were performed using a breast cancer cell line that underwent in vivo lung metastasis twice (LM2 MDA-MB-231). Furthermore, an image analysis tool that was able to enumerate and record the separating cells in real-time was developed and utilized in the analysis. By showing the ability to separate tumor cells with minimized contamination from hemocytes, we believe that the slanted weir microfluidic device possesses a high potential not only for liquid biopsy, but also for aiding future cancer research.

## 2. Results and Discussion

### 2.1. Designing the Slanted Weir Microfluidic Device

**Figure 1:**
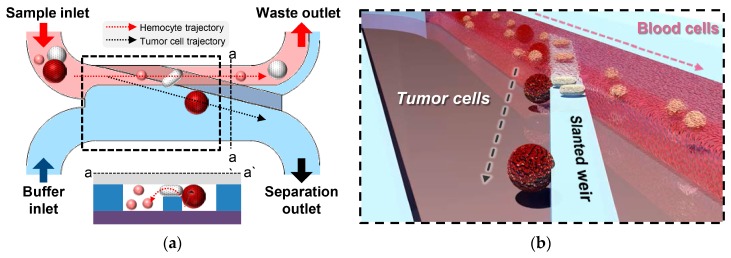
Schematic illustrations of a slanted weir microfluidic device. (**a**) An overview of the slanted weir device. (**b**) Enlarged illustration of circulating tumor cells (CTCs) being separated by the slanted weir, utilizing their distinct size and deformability.

The microfluidic device consists of two inlets for the sample and buffer injection, a slanted weir, and two outlets for waste and separated cell collection (Figure 1a). The slanted weir lies in the main channel, which indicates the part of the fluidic channel starting from the confluence of the two inlet streams until the branch point. The slanted weir extends from the upper channel wall of the main channel to the branch point, traversing the upper half of the main channel where the sample flows. The height of the slanted weir was designed to be lower than that of the fluidic channel, forming a 7-μm-gap above the weir, and the gap size was determined through our previous study [52]. The separation mechanism can be explained as follows. The buffer flow focuses on the blood sample stream, making the cells interact with the slanted weir. Hemocytes that are smaller or comparable to the weir gap would easily proceed over the weir, while larger cells would be restricted. As the weir was slanted, the larger cells would proceed along the weir, rather than be immobilized by it. While being guided by the slanted weir, these larger cells experience consistent hydraulic pressure directed across the weir, enabling the deformability-based separation. The cells with a lower degree of deformation, mostly tumor cells, would endure the pressure being transferred from the blood sample stream to the buffer stream, proceeding to the separation outlet. The cells with a much higher degree of deformability, mostly leukocytes, would deform enough to be squeezed through the gap over the weir and be swept away to the waste outlet (Figure 1b).

**Figure 2:**
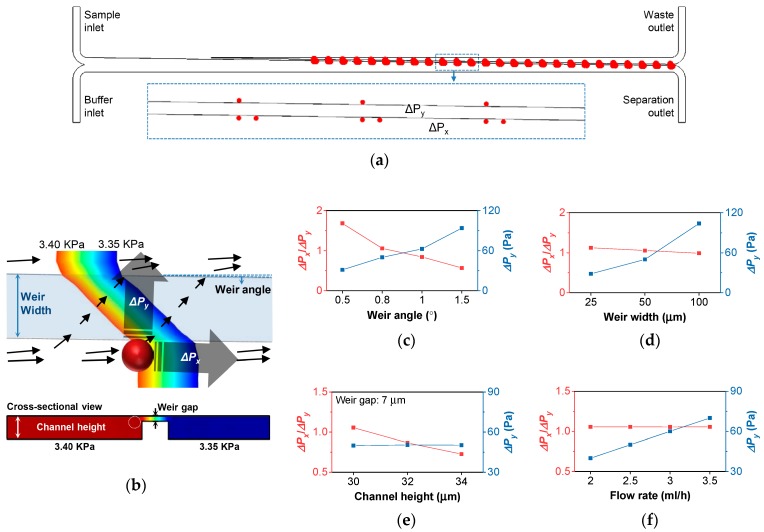
Computational analysis of the slanted weir microfluidic device. (**a**) Array of the three reference positions along the slanted weir. (**b**) Pressure distribution shown near the slanted weir. The black arrows represent streamlines. (**c**–**f**) The pressure drop ratio (*ΔP_x_*/*ΔP_y_*) (red) and the pressure drop along the slanted weir (*ΔP_x_*) (blue), according to (**c**) the weir angles, (**d**) the weir widths, (**e**) the channel heights with the fixed weir gap, and (**f**) the flow rates.

To evaluate the device performance depending on the device geometry, fluid dynamics for the various geometrical parameters were analyzed using COMSOL™ 5.2. (COMSOL Inc., Stockholm, Sweden). When a cell larger than the weir gap comes in contact with the slanted weir, its behavior should depend on the hydraulic pressure applied to the cell. Therefore, the pressure data from an array of three reference positions along the slanted weir were extracted from the simulation results so as to analyze the pressure drop along the slanted weir (*ΔP_x_*) and the pressure drop across the slanted weir (*ΔP_y_*) (Figure 2a,b). Here, the pressure drop ratio (*ΔP_x_*/*ΔP_y_*) should reflect how dominantly a cell in contact with the slanted weir should be directed along the weir. Therefore, *ΔP_x_*/*ΔP_y_* ≥ 1 should, at least, be secured to properly guide the cells along the weir. Otherwise, more pressure towards the weir gap would be applied to the cells while in contact with the weir, inducing them to become trapped in the weir gap. However, as the *ΔP_x_*/*ΔP_y_* ratio becomes higher, there would be a higher possibility of the cells flowing along the slanted weir, regardless of their size and deformability. Therefore, we expected that the slightly higher *ΔP_x_*/*ΔP_y_* ratio would be favored. Simultaneously, an optimum value of *ΔP_y_* should be provided in order to enable the cells to be separated by their deformability. If *ΔP_y_* were too low or too high, the cells would all be guided by the slanted weir, or would be forced to squeeze through, regardless of their deformability. Therefore, the optimization of *ΔP_y_* within the device would be required in order to deplete the leukocytes with a high deformability, but to keep the invasive tumor cells with a moderate deformability.

Then, to determine the device geometry, we analyzed the *ΔP_x_/ΔP_y_* ratio and *ΔP_y_* according to the various geometry conditions, namely: weir angles, weir widths, channel heights, and flow rates (Figure 2c–f). To validate our argument on the cell behavior depending on the pressure distribution near the slanted weir, the geometrical parameters should able to manipulate the *ΔP_x_*/*ΔP_y_* ratio ranging from less than to greater than one, and should able to manipulate *ΔP_y_* without disturbing the other hydrodynamic conditions. As it can be seen in the graphs, the *ΔP_x_*/*ΔP_y_* ratio depends on the weir angle, channel height, and slightly on the weir width, while *ΔP_y_* depends on the weir angle, weir width, and flow rate. However, manipulating the *ΔP_x_*/*ΔP_y_* ratio by the channel height was not favored, because inducing the higher value of the *ΔP_x_*/*ΔP_y_* ratio required lowering the channel height or enlarging the weir gap, which can hinder the cell flow or lose tumor cells. Manipulating *ΔP_y_* using the channel width was also not favored, because it affects other hydrodynamic conditions, including the *ΔP_x_*/*ΔP_y_* ratio and cell passage, making the case too complicated. Therefore, we chose the weir angle for manipulating the *ΔP_x_*/*ΔP_y_* ratio and the flow rate for manipulating *ΔP_y_* in further device validation.

### 2.2. Demonstration Using the Cancer Cell Line

To validate our arguments, we made experiments on the slanted weir devices using LM2 MDA-MB-231 breast cancer cells. They express the CD44+/CD24− phenotype, which is considered as a cancer stem cell (CSC)-like population [54,57]. CSC is known as having the ability for self-renewal as well as tumor initiation, progression, therapy resistance, and recurrence [58,59]. In breast cancer, it is also reported that CSC is related to EMT [11,60], making the tumor cells more flexible. Those tumor cells with a high metastatic potential and deformability were what we were willing to separate from the hemocytes with minimum loss. 

Then, 10^4^ tumor cells in 1 mL of 1 × Phosphate-buffered saline were introduced into the devices, and the number of tumor cells from each outlet were compared to analyze the separation efficiency. The weir angles were fabricated at 0.5°, 0.8°, and 1° to achieve a *ΔP_x_*/*ΔP_y_* ratio of 1.7, 1.1, and 0.8, respectively. In addition, *ΔP_y_* was tested for 40, 50, 60, and 70 Pa. The flow rate ratio between the sample and buffer stream was determined so as to assure that the sample stream flows over the weir, inducing all of the tumor cells to experience the slanted weir. Through the preliminary experiment using a blood sample, it was confirmed that the sample-to-buffer flow rate ratio must be 1:4 or with a higher portion of the buffer flow rate. 

**Figure 3:**
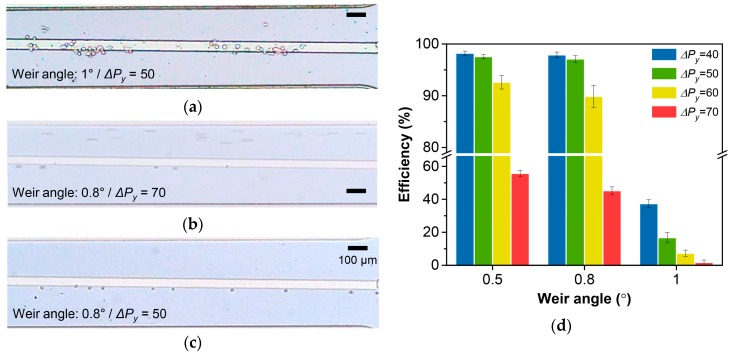
Demonstration using the cancer cell line. (**a**–**c**) Optical microscope images of tumor cell separation using (**a**) the weir angle of 1° and *ΔP_y_* of 50 Pa, (**b**) the weir angle of 0.8° and *ΔP_y_* of 70 Pa, and (**c**) the weir angle of 0.8° and *ΔP_y_* of 50 Pa. (**d**) Separation efficiency according to the weir angles and *ΔP_y_*.

We first briefly tested the effect of the *ΔP_x_*/*ΔP_y_* ratio and *ΔP_y_* on the separation efficiency (Figure 3a–c, see Supplementary Video S1). The tumor cells that encountered the 1° weir (*ΔP_x_*/*ΔP_y_* = 0.8), with the flow rate that refers to *ΔP_y_* of 50 Pa, were mostly trapped in the weir gap or flowed over the weir (Figure 3a). Also, the tumor cells that encountered the 0.8° weir (*ΔP_x_*/*ΔP_y_* = 1.1) with the flow rate that refers to *ΔP_y_* of 70 Pa, mostly flowed over the weir (Figure 3b). Meanwhile, the tumor cells that encountered the 0.8° weir (*ΔP_x_*/*ΔP_y_* = 1.1) with the flow rate that refers to a *ΔP_y_* of 50 Pa, efficiently followed the slanted weir (Figure 3c). 

The quantitative result according to the *ΔP_x_*/*ΔP_y_* ratio and *ΔP_y_* is shown in Figure 3d. While with the favorable *ΔP_y_*, the weir angles that produced *ΔP_x_*/*ΔP_y_* > 1 (0.5° and 0.8°) resulted in very high separation efficiencies, showing a maximum value of 98.9% (Figure S1). In both cases, we did not observe tumor cells that were trapped within the devices. The weir angle of 1° with a *ΔP_x_*/*ΔP_y_* ratio of 0.8, resulted in a poor separation efficiency with a maximum value of 39.7%. The increase of the weir angle by just 0.2° induced a relatively higher pressure towards the weir than along it, which forced the tumor cells to deform and get trapped in the weir gap, even when the *ΔP_y_* was low enough. On the other hand, even with the favorable *ΔP_x_*/*ΔP_y_* ratio, the average separation efficiency slightly decreased from 97.6% to 92.6% for the 0.5° weir, and from 97.1% to 89.9% for the 0.8° weir, when *ΔP_y_* increased from 50 Pa to 60 Pa. When *ΔP_y_* increased to 70 Pa, the separation efficiency drastically decreased by ~40% points for both 0.5° and 0.8°.

Then, we analyzed the effect of the sample-to-buffer flow rate ratio on the separation efficiency. The total flow rate was set at 2.5 mL/h for the 0.8° weir and at 3.8 mL/h for the 0.5° weir, which produced a *ΔP_y_* of 50 Pa, and the sample-to-buffer flow rate ratios of 1:4 and 1:5 were tested, with no significant difference found. Furthermore, the viability of the collected tumor cells was tested by the trypan blue assay, which showed 97.1% for the 0.8° weir and 95.8% for the 0.5° weir (Figure S2). The slightly lower viability shown from the 0.5° weir device might be due to the higher shear rate induced by the higher total flow rate.

From the quantitative analysis, we decided to use a 0.5° and 0.8° weir with the flow rate inducing *ΔP_y_* of 50 Pa in the following experiments. The reason for choosing 50 Pa over 40 Pa, was to better utilize the cell deformability. Both of the conditions showed a high separation efficiency (>97%) using LM2 MDA-MB-231, and it was also shown that the higher *ΔP_y_* pushes the cells more towards the weir gap. Therefore, the condition showing *ΔP_y_* of 50 Pa can better separate the tumor cells from the leukocytes by means of the differing degrees of deformability.

### 2.3. Image Analysis of the Cancer Cell Line

The image analysis tool was realized using the Gaussian mixture model, which adaptively modeled the background to track and analyze the moving objects (see supplementary for detail). The image analysis tool was able to provide the number, size, and aspect ratio of all of the particles passing the region of interests (ROIs), as well as their videos. This enabled the precise evaluation of the separation efficiency for low cell concentrations and the image-based analysis of the cells during their separation.

**Figure 4:**
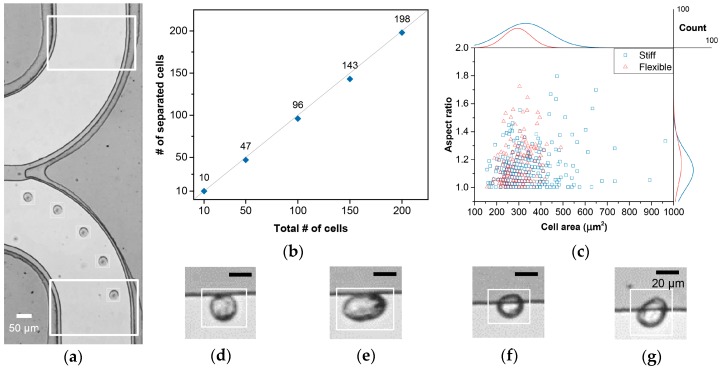
Analysis using the image analysis tool. (**a**) Time-lapse image of a tumor cell proceeding to the separation outlet. The larger rectangles indicate region of interests (ROIs) of the two outlets, and the smaller rectangles indicate a cell detection. (**b**) The number of separated cells according to the total number of detected cells from both outlets. (**c**) Distribution of the LM2 MDA-MB-231 cells (n = 503) by their size, aspect ratio, and differing deformability. The line graphs at the top and the right sides refer to the cell count distributions. (**d**–**g**) Optical images showing the various physical properties of the separating tumor cells, namely: (**d**) stiff and spherical, (**e**) stiff and elongated, (**f**) flexible and spherical, and (**g**) flexible and elongated. Tool bars in Figure 4d–g represent 20 μm.

To quantitatively analyze the device performance for low cell concentrations, ROIs were designated at the passages to each outlet (Figure 4a and Figure S3, see Supplementary Video S2). Also, the tumor cell suspension samples with an average cell concentration of 100 cell/ml were applied to the device with a 0.8° weir and a *ΔP_y_* of 50 Pa. For a combined total of 10, 50, 100, 150, and 200 tumor cells perceived at both outlet ROIs, the number of tumor cells spotted at the waste outlet ROI were 0, 3, 4, 7, and 2, respectively, giving an average 96.9% separation efficiency (Figure 4b). The size distribution of the tumor cells from each outlet was also analyzed (Figure S4). For the separated cells, the peak cell population appeared to have an area of 300–350 μm^2^, and doublets were observed starting from ~560 μm^2^. Wasted cells, on the other hand, did not show an evident peak cell area population, with no cell larger than 350 μm^2^. However, as enumerated above, the number of wasted tumor cells varied throughout the trials, and tumor cells under 350 μm^2^ were found from both outlets. This variation might be due to a variation in *ΔP_y_* caused by weir gap fluctuations per device, owing to fabrication accuracy limitations, or the difference in the initial position of the tumor cells, which decides how long the cells are affected by the pressure across the weir. 

We further investigated the videos of the LM2 MDA-MB-231 cells taken during separation, and monitored their interaction with the slanted weir to characterize the physical properties of the LM2 MDA-MB-231 cells (Figure 4c). Firstly, different morphologies of the tumor cells were observed. While most tumor cells showed a spherical shape, several tumor cells showed elongated shapes (Figure 4d,e). We were also able to observe differing deformation characteristics of tumor cells as they experienced the slanted weir. Relatively flexible tumor cells were observed as being partially squeezed into the weir gap, while still being separated and guided to the separation outlet (Figure 4f,g). We expect that the differing separation feature might be due to the differing flexibility of a cytoplasm. The key factor, that the partially squeezed cells did not pass through weir gap, might be due to the stiff and large nucleus that endured the hydraulic pressure.

### 2.4. Separation of Spiked Tumor Cells from Whole Blood

**Figure 5:**
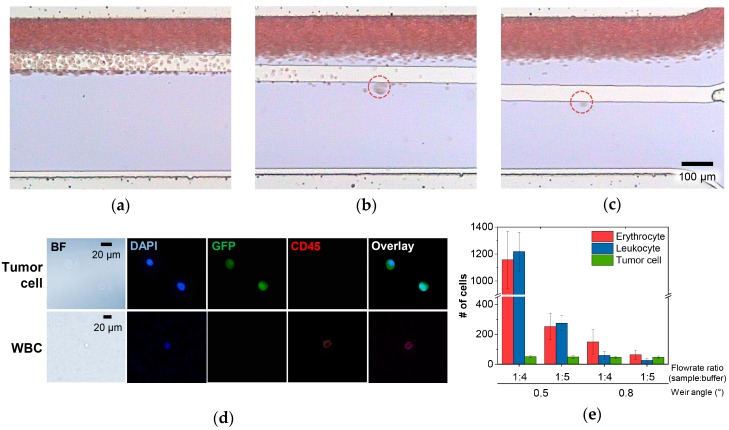
Demonstration using the tumor cell spiked whole blood samples. (**a**–**c**) Optical images of tumor cell separation from three different regions according to distances from the starting point of slanted weir, namely: (**a**) 10 mm, (**b**) 14 mm, and (**c**) 18 mm. (**d**) Bright field and fluorescence images of the separated tumor cells and a leukocyte. (**e**) The number of separated cells according to the weir angles and flow rate ratios.

With the basic performance of the device demonstrated, the whole blood processing capability was tested by applying 2 mL of unprocessed whole blood spiked with LM2 MDA-MB-231 (~25 cells/mL). Figure 5a–c shows the optical images of the separating cell from the blood sample stream by the slanted weir, from three different positions where hemocytes were seen to flow over the weir to the waste outlet (see Supplementary Video S3). The hemocytes were mostly removed by about 14 mm from the starting point of the slanted weir, allowing the large cells expected as tumor cells to roll along the weir at 18 mm. The image analysis tool also verified that the separating cells showed similar features as seen in Figure 4d–g (see Supplementary Video S4).

To evaluate the device performance, we examined the separated cells via immunofluorescence, and analyzed the number of separated cells, depending on weir angles (0.5° and 0.8°) and sample-to-buffer flow rate ratios (1:4 and ~1:5), while *ΔP_y_* was fixed at 50 Pa (Figure 5d–e). The number of separated tumor cells were consistent in all of the conditions with a ~97% separation efficiency, which followed the results presented earlier. However, a distinct condition was shown, achieving high hemocyte depletion that refers to the high purity of the separated tumor cells.

The number of separated hemocytes, both erythrocytes and leukocytes, decreased as the flow rate ratio changed from 1:4 to 1:5 for all of the tested weir angles. The higher buffer flow rate enhanced the focusing of the blood sample stream, allowing the hemocytes, especially those on the boundary between the two, to have a higher chance of traversing over the weir, as the *ΔP_x_*/*ΔP_y_* ratio was greater than one. When the weir angle was changed from 0.5° to 0.8°, we also observed an effective reduction of the separated hemocytes. Interestingly, the enumerated leukocytes from the total enumerated hemocytes was ~29% for the 0.8° weir, while that of the 0.5° weir showed ~50%. This indicates that a more effective depletion of leukocytes was achievable using the 0.8° weir compared with the 0.5° weir. As previously expected, this might be due to the higher proportion of *ΔP_y_* in the *ΔP_x_*/*ΔP_y_* ratio shown in the 0.8° weir than the 0.5° weir, making the cells approaching the weir more affected by the hydraulic pressure towards the weir. In conclusion, an average of 63 erythrocytes and 26 leukocytes were separated using the optimized condition, which indicates a ~8 log depletion of erythrocytes and ~5.6 log depletion of leukocytes, considering that as much as 5 × 10^9^ erythrocytes and 5 × 10^6^ leukocytes exist per 1 mL of blood on average. The depletion of the hemocytes was explored further so as to discover how the hemocytes that were supposed to flow over the weir were retrieved from the separation outlet. From the videos provided by the image analysis tool, we found the hemocytes being dragged to the separation outlet along in fibrin clot or in the skewed flow stream formed by large thrombus, microemboli, or bubbles (Figure S5). Nevertheless, the hemocyte depletion rate achieved, along with the high separation efficiency of the LM2 MDA-MB-231 cells, is still a noteworthy result, considering the deformability of the utilized cancer cell line. We believe that the key factor for this achievement lies in the well-designed pressure distribution near the slanted weir, allowing the tumor cells, although flexible but with a large and stiff nucleus, to flow along the slanted weir while inducing the leukocytes, although large but with a flexible small nucleus, to pass through the weir gap.

## 3. Materials and Methods

### 3.1. Device Fabrication

The microfluidic device was realized on a 4.5 × 4.5 cm^2^ surface oxidized silicon chip. Double-layer photolithography was used to pattern the slanted weir integrated microfluidic channel (Figure S6). The first layer was spin-coated with a SU-8 2050 (Microchem, Westborough, MA, USA) photoresist, which was 23 μm thick, and the weir structure was patterned. The second layer, which defines the weir gap, was spin-coated with the SU-8 2007 (Microchem) photoresist, which was 7 μm thick. The main channel was 500 μm, while its length varied according to the weir angle. For encapsulation, the patterned SU-8 was exposed to O_2_ plasma (35 W, 100 mTorr, 30 s), followed by dipping in 5% (3-Aminopropyl)triethoxysilane for 10 min at 80 °C, rinsing with distilled water, and blow drying with N_2_. It was then attached with the O_2_ plasma (35 W, 100 mTorr, 30 s) treated poly(dimethylsiloxane) that had inlet and outlet holes on a 70 °C hot plate for 10 min, with a 500 g weight applied on the top.

### 3.2. Tumor Cell Preparation

The human breast cancer cell line MDA-MB-231 was established from the American Type Culture Collection (Manassas, VA, USA). The cells were maintained at 37 °C with 5% CO_2_ in Dulbecco’s modified eagle medium (DMEM; Thermo Fisher Scientific, Waltham, MA, USA), supplemented with 10% fetal bovine serum (FBS), penicillin (100 U/mL), and streptomycin (100 μg/mL). By tail vein injection into athymic nude mice, the MDA-MB-231 cells were metastasized to the lung, and the lung metastasized MDA-MB-231 cells were named LM1. Likewise, the LM1 MDA-MB-231 cells were injected into the mouse tail vein and metastasized again to the lung. Because those cells were metastasized twice on the lung, they were designated as LM2 MDA-MB-231 cells.

### 3.3. Animal Experiments

MDA-MB-231 (1 × 10^6^ cells/200 μL) cells that were transduced with green fluorescent protein (MSCV-IRES-eGFP) were injected into the tail vein of athymic nude mice (n = 4; Orient, Seongnam, Korea). Whole lungs were surgically taken and fixed with 4% paraformaldehyde after the mice were euthanized with CO_2_ gas. The lung metastasis was analyzed by counting the number of foci on the lung surface under a microscope. This study was reviewed and approved by the Institutional Animal Care and Use Committee of Center for Laboratory Animal Sciences, Medical Research Coordinating Center, HYU Industry-University Cooperation Foundation (2015-0180A).

### 3.4. Device Operation

Two syringe pumps (Chemyx, Stafford, TX, USA) were used to inject a sample and buffer (Figure S7). Prior to the sample injection, the microfluidic device was filled with 0.2% Pluronic F-127 (Sigma Aldrich, St. Louis, MO, USA) for 30 min, so as to prevent the non-specific adsorption of cells. A custom-made syringe rocker was used for the sample injection so as to prevent cell sedimentation in the syringe (Figure S8). The separation of the tumor cells was observed under a microscope with a high-speed camera (Vision Research, Wayne, NJ, USA), while another complementary metal-oxide-semiconductor (CMOS) camera (Toshiba, Tokyo, Japan) was used for the image analysis application. The processed sample was collected in 15 mL conical tubes.

### 3.5. Whole Blood Processing and Immunofluorescence

Written informed consent was obtained from healthy donors at Hanyang University Seoul Hospital, with approval granted by the Hanyang University Institutional Review Board (HYI-17-057-3). All of the methods were carried out in accordance with the approved guidelines. The blood samples were processed within five hours of collection. The collected samples from the separation outlet were centrifuged for 5 min at 200 g on poly-l-lysine coated substrates. For the tumor cell spiked whole blood experiments, staining was performed with phycoerythrin (PE) conjugated anti-CD45 (Southern Biotech, Birmingham, AL, USA), followed by counter staining with 4,6-diamidino-2-phenylindole (DAPI; Vector Labs, Burlingame, CA, USA). 

## 4. Conclusions

We have developed and demonstrated the slanted weir microfluidic device that was able to efficiently separate tumor cells, with minimized contamination from hemocytes. Well-organized pressure distribution near the slanted weir enabled the precise utilization of the cell deformability in addition to the size-based separation. The microfluidic device was demonstrated using the invasive breast cancer cell line (LM2 MDA-MB-231), achieving a high separation efficiency (~97%) with minimum contamination from the hemocytes, showing an 8 log depletion of erythrocytes and a 5.6 log depletion of leukocytes from 2 mL of unprocessed whole blood. The morphology and the differing deformability of the separating cells were also analyzed using the image analysis tool, which provided automatic, real-time enumeration and recording of the separated cells. The image analysis tool verified that the microfluidic device maintained a high separation efficiency, even in the sample condition of a low cell concentration, and the existence of various morphologies with differing deformability within the tumor cells that were able to be separated.

Improvement in the throughput by device parallelization is currently in progress, and clinical tests are planned for future experiments. As our microfluidic device was optimized using the invasive cancer cell line, we hopefully expect the successful separation of CTCs from cancer patients, including the tumor initiating subpopulation, such as CSCs. With further development, the slanted weir microfluidic system may be applied to draw valuable clinical information, contributing to prognosis and possible personalized therapy for cancer patients.

## Supplementary Materials

The following are available online at https://www.mdpi.com/2072-6694/11/2/200/s1, Document describing details of the image analysis tool; Figure S1: Optical images of the collected tumor cells; Figure S2: Images of a trypan blue assay; Figure S3: Introduction of the image analysis tool; Figure S4: Size distribution of LM2 MDA-MB-231 cells; Figure S5: Images of hemocytes flowing to the separation outlet affected by various large objects; Figure S6: Images of the fabricated slanted weir microfluidic device; Figure S7: Photograph of the experimental set-up; Figure S8: Photographs of the 3D-printed syringe rocker; Video S1: Effect of pressure distribution on tumor cell separation; Video S2: Demonstration of the image analysis tool; Video S3: Demonstration of tumor cell separation from whole blood; Video S4: Image analysis in tumor cell spiked whole blood samples.
