# Supplementary material for: Continuous Separation of Circulating Tumor Cells from Whole Blood Using a Slanted Weir Microfluidic Device
Source: Cancers (Basel). 2019 Feb 10;11(2):200. doi: 10.3390/cancers11020200 (PMC6406949; doi:10.3390/cancers11020200)
Supplement: Supplementary file 1 [file cancers-11-00200-s001.zip › cancers-420395-supplement-final/cancers-420395 supplementary.pdf]

Continuous Separation of Circulating Tumor Cells from Whole Blood using a Slanted Weir Microfluidic Device

Supplementary Material

Image analysis tool

The tool utilized a CMOS camera (Toshiba, Tokyo, Japan) with 300 fps for capturing the microfluidic device. The image analysis consisted of three stages: background subtraction, multi-object tracking, and enumeration. In the background subtraction stage, static background of the microfluidic device was removed from the scene and only moving objects remained in the image. To adaptively model the background, Gaussian mixture model was utilized [1,2]. In the multi-object tracking stage, detected objects over several frames were associated to get trajectories. The association was based on the predicted positions of the objects calculated by using positions of previous three frames. In the enumeration stage, the number of detected objects passing through a region of interest was counted. To assist the verification of tumor cell candidates, the image analysis tool provided moving images of every enumerated object as well as the object size, the length of major axis and minor axis, and the aspect ratio.

References

1. Zivkovic, Z. Improved adaptive Gaussian mixture model for background subtraction. *Proc. 17th Int. Conf. on Pattern Recognition (ICPR'04, IEEE Computer Soc.)* **2004**, *2*, 28–31.
2. Zivkovic, Z.; van der Heijden, F. Efficient adaptive density estimation per image pixel for the task of background subtraction. *Pattern Recogn. Lett.* **2006**, *27*, 773–780.

Supplementary Figures

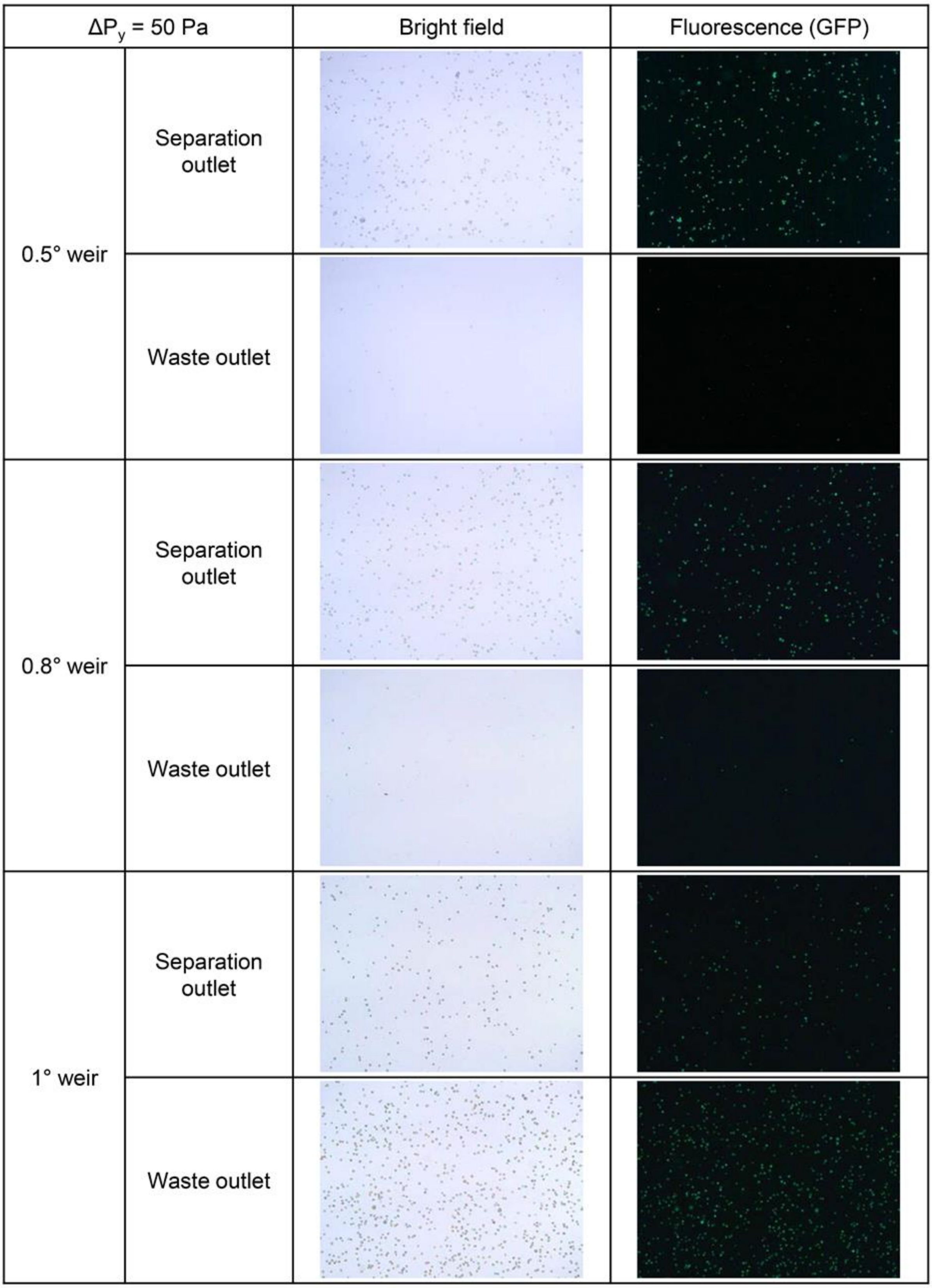

Figure S1. Optical images of the collected tumor cells from the devices having the weir angles of 0.5°, 0.8°, 1° when ΔP_y was set at 50 Pa.

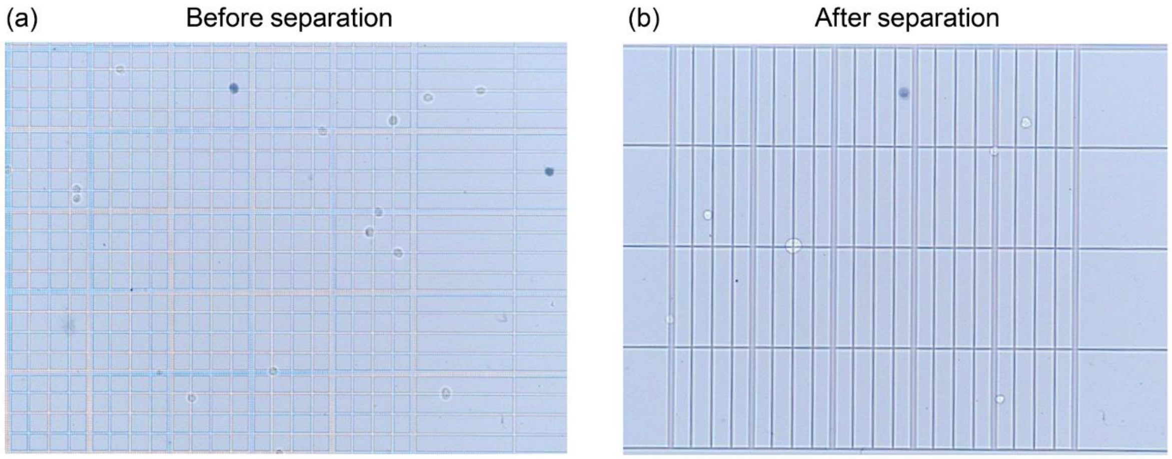

Figure S2. Images of a trypan blue assay; (**a**,**b**) Viability of LM2 MDA-MB-231 cells (**a**) before the separation and (**b**) after the separation using the 0.8° weir and ΔP_y of 50 Pa.

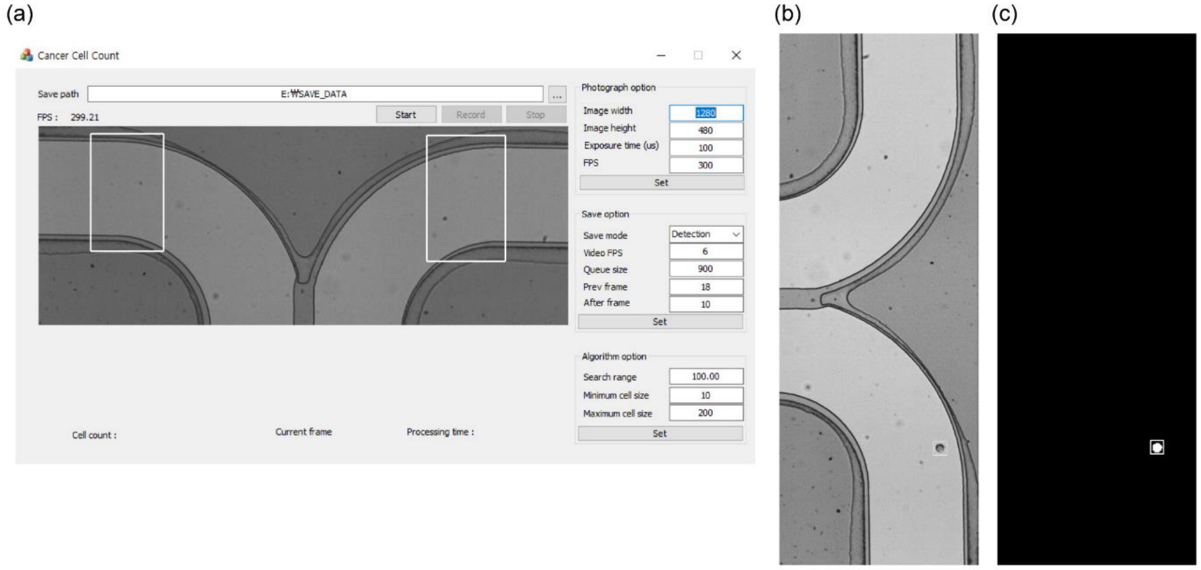

Figure S3. Introduction of the image analysis tool; (**a**) User interface of the image analysis tool. It provides following real-time information to users: the number of counted tumor cells, the number of processed frames, current FPS, and running time; (**b**) Image showing the detection of a tumor cell (white square); (**c**) Image showing how the software recognizes the cell.

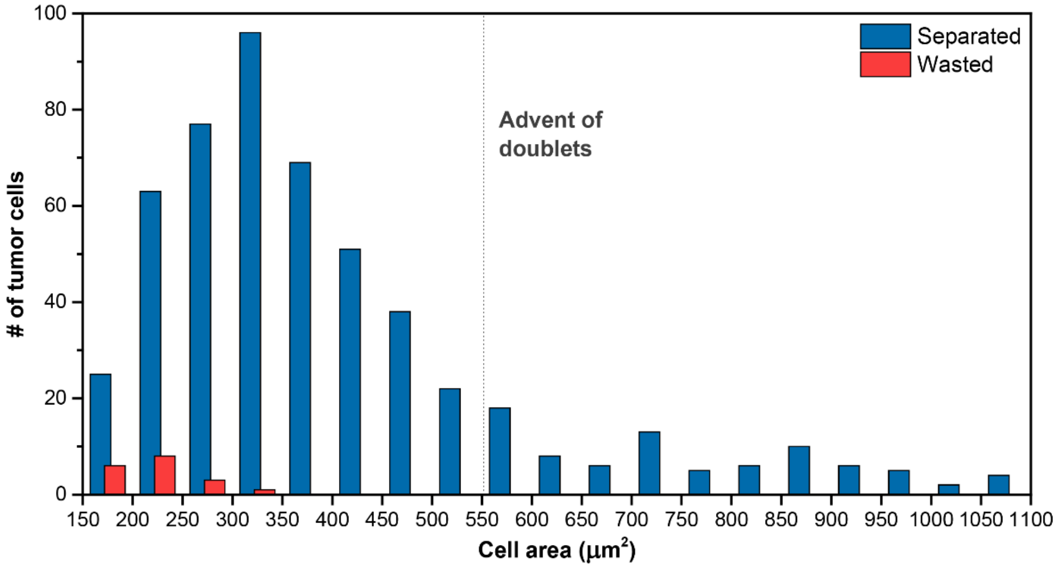

Figure S4. Size distribution of LM2 MDA-MB-231 cells perceived at both separation and waste outlets.

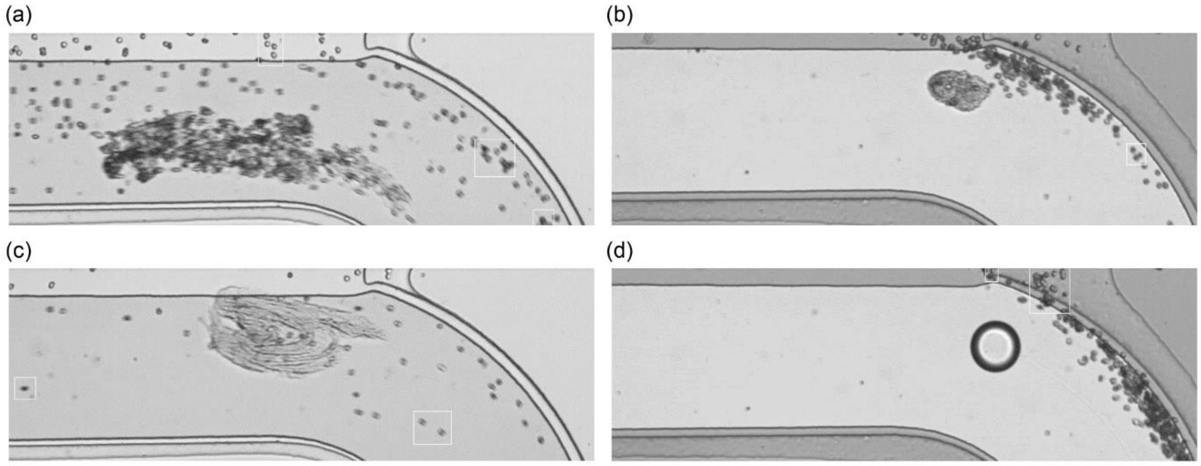

Figure S5. Images of hemocytes flowing to the separation outlet affected by various large objects; (**a**–**c**) blood clots; (**d**) a bubble.

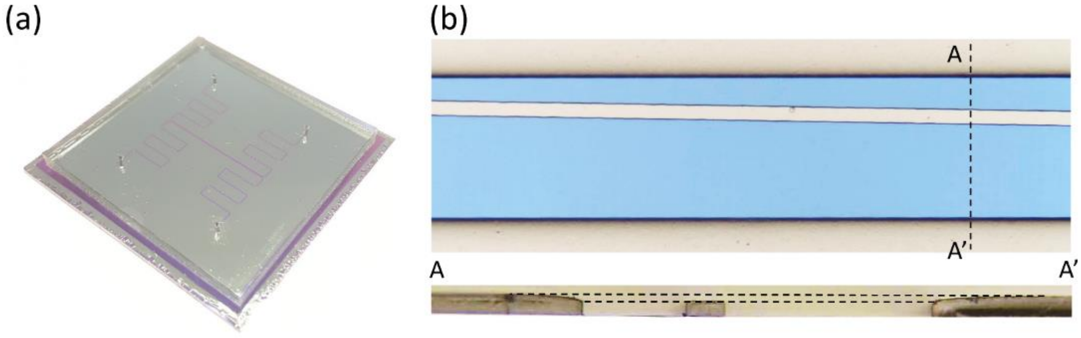

Figure S6. Images of the fabricated slanted weir microfluidic device; (**a**) Photograph of a fabricated device; (**b**) Microscopic images showing the top view and the cross-sectional view of the device prior to channel sealing.

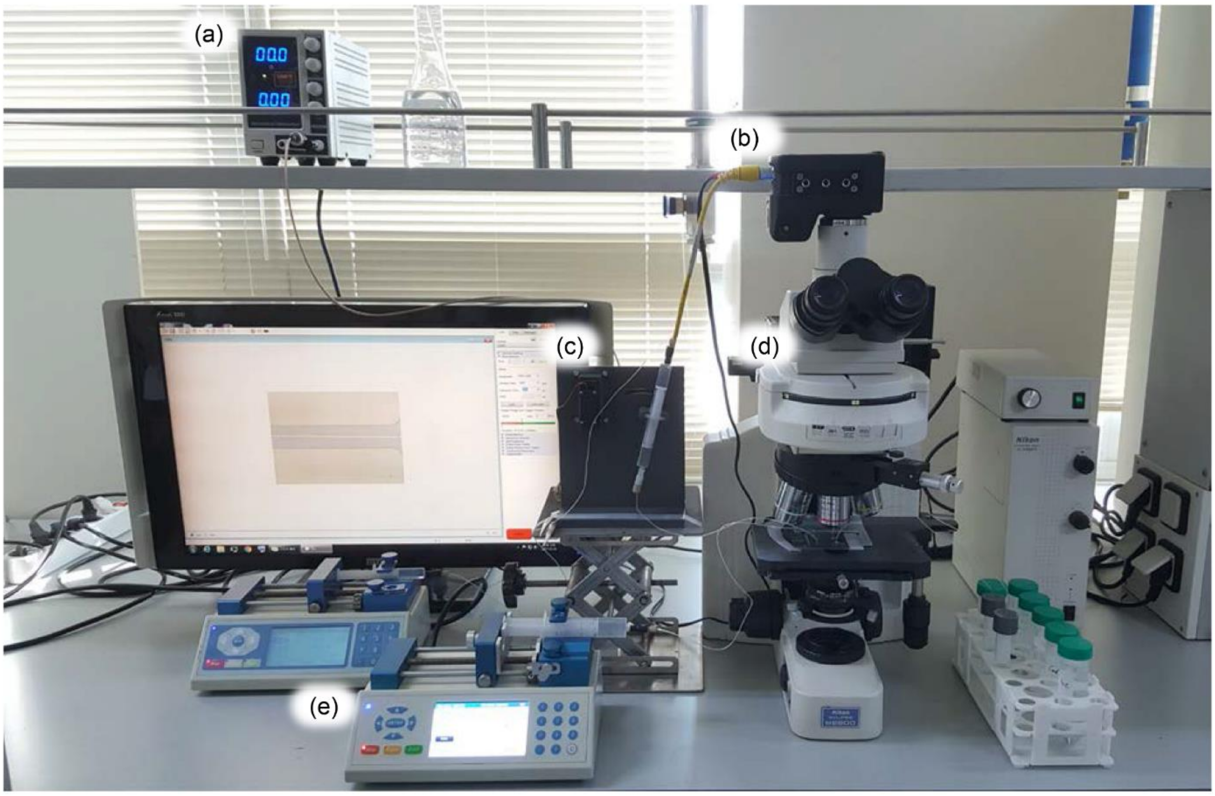

(a) DC power supply (b) High speed camera (c) 3D printed syringe rocker
(d) Microscope (e) Syringe pumps

Figure S7. Photograph of the experimental set-up.

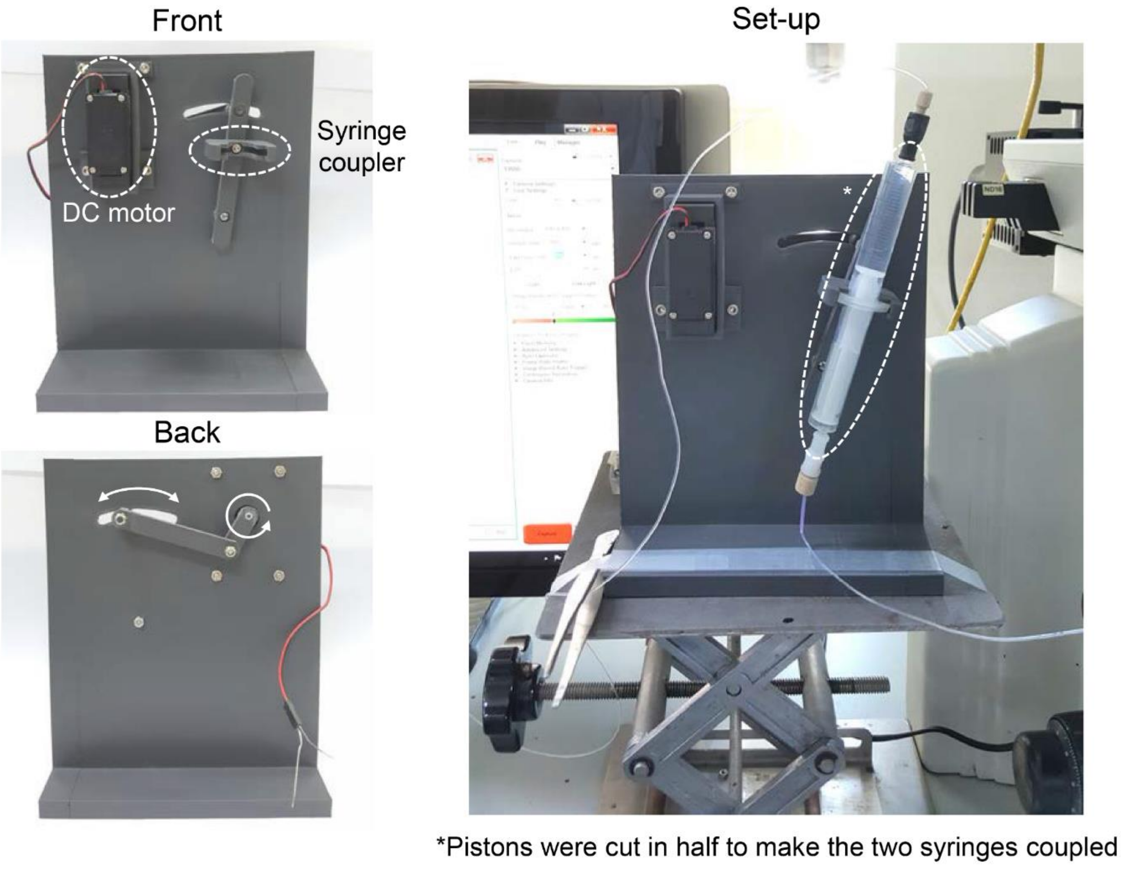

Figure S8. Photographs of the 3D-printed syringe rocker made to prevent cell sedimentation.

Supplementary Videos

Supplementary video S1. This video shows the effect of pressure distribution on separation efficiency. The optimized device was achieved by using a 0.8° weir ($\Delta P_x/\Delta P_y = 1.1$) and flowrate of 2.5 ml/h ($\Delta P_y = 50$ Pa). When the flowrate was increased to 3.0 ml/h (ΔP_y increased to 60 Pa), some cells flowed over the weir proceeding to the waste channel. When the weir angle was increased to 1° ($\Delta P_x/\Delta P_y$ decreased to 0.8), most cells were observed being trapped in the gap on top of the weir.

Supplementary video S2. This video shows a demonstration of the image analysis tool operation for low cell concentration analysis. The observing region was set to enable cell cognition from both outlets. Five cells in a row were perceived from both outlet ROIs by the image analysis tool, and all of them were observed proceeding to the separation outlet.

Supplementary video S3. This video shows the operation of the slanted weir device when a tumor cell spiked blood sample was applied. Three different positions were observed to analyze the cell behavior at the slanted weir. At the position 10 mm from the starting point of slanted weir, most hemocytes were observed flowing over the weir nevertheless, numerous hemocytes still flowed along the weir. At the position 14 mm from the starting point of slanted weir, a cancer cell appeared flowing along the weir while fewer hemocytes were observed. At the position 18 mm from the starting point of slanted weir, a cancer cell was observed flowing to the separation outlet, while all hemocytes flowed to the waste outlet.

Supplementary video S4. This video shows differing morphology and deformability of the cells separated from the tumor cell spiked whole blood samples. The observing region was set to recognize cells interacting with the slanted weir and proceeding to the separation outlet. A stiff and circular cell, a flexible and circular cell, a stiff and elongated cell, and a flexible and elongated cell were demonstrated.
